# Effective Drug Delivery in Diffuse Intrinsic Pontine Glioma: A Theoretical Model to Identify Potential Candidates

**DOI:** 10.3389/fonc.2017.00254

**Published:** 2017-10-30

**Authors:** Fatma E. El-Khouly, Dannis G. van Vuurden, Thom Stroink, Esther Hulleman, Gertjan J. L. Kaspers, N. Harry Hendrikse, Sophie E. M. Veldhuijzen van Zanten

**Affiliations:** ^1^Department of Pediatric Oncology – Hematology, VU University Medical Center, Amsterdam, Netherlands; ^2^Department of Clinical Pharmacology and Pharmacy, VU University Medical Center, Amsterdam, Netherlands; ^3^Department of Pharmaceutical Sciences, Utrecht University, Utrecht, Netherlands; ^4^Princess Máxima Center for Pediatric Oncology, Utrecht, Netherlands; ^5^Department of Radiology and Nuclear Medicine, VU University Medical Center, Amsterdam, Netherlands

**Keywords:** diffuse intrinsic pontine glioma, blood–brain barrier, chemotherapy, convection-enhanced delivery, drug delivery

## Abstract

Despite decades of clinical trials for diffuse intrinsic pontine glioma (DIPG), patient survival does not exceed 10% at two years post-diagnosis. Lack of benefit from systemic chemotherapy may be attributed to an intact bloodbrain barrier (BBB). We aim to develop a theoretical model including relevant physicochemical properties in order to review whether applied chemotherapeutics are suitable for passive diffusion through an intact BBB or whether local administration *via* convection-enhanced delivery (CED) may increase their therapeutic potential. Physicochemical properties (lipophilicity, molecular weight, and charge in physiological environment) of anticancer drugs historically and currently administered to DIPG patients, that affect passive diffusion over the BBB, were included in the model. Subsequently, the likelihood of BBB passage of these drugs was ascertained, as well as their potential for intratumoral administration *via* CED. As only non-molecularly charged, lipophilic, and relatively small sized drugs are likely to passively diffuse through the BBB, out of 51 drugs modeled, only 8 (15%)—carmustine, lomustine, erlotinib, vismodegib, lenalomide, thalidomide, vorinostat, and mebendazole—are theoretically qualified for systemic administration in DIPG. Local administration *via* CED might create more therapeutic options, excluding only positively charged drugs and drugs that are either prodrugs and/or only available as oral formulation. A wide variety of drugs have been administered systemically to DIPG patients. Our model shows that only few are likely to penetrate the BBB *via* passive diffusion, which may partly explain the lack of efficacy. Drug distribution *via* CED is less dependent on physicochemical properties and may increase the therapeutic options for DIPG.

## Background

Diffuse intrinsic pontine glioma (DIPG) is a rare, aggressive childhood malignancy of the brainstem with a 2-year survival rate of 10% (1, 2). Unlike the spectacular increase in survival of childhood leukemia patients from <10% to over 80% in the last 50 years, the prospect for patients suffering from DIPG has not changed (3–5), likely due to a lack of success from (chemo)therapeutic strategies. Historically, this was blamed on the supposed resistance of DIPG tumor cells to cytotoxic agents. Preclinical studies have, however, recently shown that primary cultures derived from DIPG patients, are actually not resistant to a number of traditionally used cytotoxic drugs and novel targeted chemotherapeutics (5, 6). These results from pre-clinical studies contrasting with disappointing outcomes of clinical studies led to a shifting paradigm toward the hypothesis of a possible delivery problem of chemotherapeutics over the blood-brain barrier (BBB). This supposedly prevents drugs from reaching the tumor properly (7). In many brain tumors, the integrity of the BBB is affected by the formation of disordered and highly permeable tumor neovasculature. Infiltrating tumors, such as DIPG, however, make use of the existing brain vasculature with normal BBB integrity (8). Noteworthy in this respect, especially early-disease DIPGs show limited contrast enhancement after intravenous administration of gadolinium compared to glioblastoma multiforme tumors that harbor highly neovascular regions. As gadolinium has an average molecular weight of 545 kDa, which largely exceeds the penetration cut-off of the BBB (e.g., 400–600 Da), limited contrast enhancement in these tumors is suggestive of a largely intact BBB (9–11). Furthermore, to illustrate, studies investigating biopsy-derived intratumoral drug concentrations of systemically delivered drugs in adults with high-grade glioma show low, potentially sub-therapeutic local drug concentrations, especially in non-enhancing tumor regions (12–14).

To overcome the BBB, novel drug delivery techniques, such as convection-enhanced delivery (CED), have been developed. With CED, chemotherapeutic agents are administered directly into the tumor microenvironment *via* a highly controlled positive pressure gradient and a constant flow induced by a pump. This enables homogeneous distribution of high drug concentrations over an easy-to-define distance, presumably increasing the therapeutic potential and avoiding systemic toxicities (14, 15).

In this study, we aim to review all chemotherapeutic drugs (previously) administered systemically to DIPG patients by means of a theoretical model including all physicochemical properties that influence passive diffusion, to indicate their likeliness of passage over an intact BBB in DIPG. Furthermore, we aim to indicate whether local administration of these drugs *via* convection-enhanced delivery (CED) may increase their therapeutic potential.

## Model Design

An extensive search of the literature and trial databases was performed to identify all chemotherapeutics historically employed in DIPG patients. Databases of Medline/PubMed and The Cochrane Library were searched for potentially relevant articles. The search strategy combined controlled and free text words for the target population (e.g., children), the tumor type (e.g., DIPG or pontine glioma) and the application of chemotherapeutic drugs. The reference lists of all included articles were searched for additional studies. In addition, trial registries (www.clinicaltrials.gov, www.clinicaltrialsregister.eu) as well as websites from consortia treating children with brain tumors (www.itcc-consortium.org, www.pbtc.org, www.childrensoncologygroup.org) were searched for clinical trials in DIPG. The complete search strategy can be found in the supplementary data.

Subsequently, drug simulations were performed for both systemic administration to predict passive diffusion over an intact BBB and for local intratumoral drug delivery *via* CED, to predict convection-distribution efficacy, using relevant physicochemical properties (molecular weight, lipophilicity, and molecular charge). To this aim, physicochemical property data were extracted from various chemical databases: PubChem chemistry database, Drugbank.ca and Clarke’s Analysis of Drugs and Poisons. The molecular charge in physiologic environment was simulated by MarvinSketch^®^ (Chemaxon), an advanced chemical editor for drawing chemical structures and calculating basic physicochemical properties (e.g., molecular charge, log *P*), using specific algorithms.

### Drug Simulation for Systemic Drug Administration

The BBB, formed by tightly interconnected endothelial cells in the capillary walls of the brain vasculature, protects the brain by limiting the inter- and paracellular transport of foreign substances from the systemic blood flow (16). Systemically applied chemotherapeutics enter the brain *via passive diffusion* or *active transport mechanisms*. The balance between in- and outflow depends both on the physicochemical properties of the drug itself and on its affinity for drug in- and efflux transporters and receptors expressed in the BBB.

The Lipinski Rule of 5 is used to determine a drug’s permeability. According to this rule good permeability is likely if: (i) the molecular weight is ≤500 Da, (ii) the lipophilicity, measured by the partition coefficient (log *P*), is ≤5 (optimal value of 2.0–3.5), (iii) the structure has no more than 5 hydrogen bond donors, and (iv) no more than 10 hydrogen bond donor acceptors (16–18). Taking these rules into consideration, the physicochemical properties that determine passive diffusion through the BBB, are molecular weight, lipophilicity, and molecular charge. For every drug, *molecular weight* and *Log P* were included in the model and subsequently, the chemical structure was used to simulate the *molecular charge* of a drug in physiological environment (pH 7.4). Drugs with a molecular (positive or negative) charge of ≤10% were considered to be able to passively diffuse through the BBB. Drugs with a (positive or negative) molecular charge of ≥90% are considered to have a higher affinity for the hydrophilic environment of the blood and are therefore not likely to passively diffuse through the BBB. Drugs with a molecular charge between 10 and 90% are partly able to diffuse through the BBB, but are likely not to reach their therapeutic concentration after systemic administration. In case of prodrugs (i.e., inactive compounds that require metabolization into a pharmacologically active form), the physicochemical properties of the active metabolites were evaluated in the model.

### Drug Simulation for Local Administration *via* CED

For CED, drug distribution over the tumor volume mainly depends on two determinants: positive pressure gradient created by the drug infusion system and the *molecular charge* of a drug: positively charged molecules tend to form complexes with negatively charged cell membrane components, leading to lower distribution volumes (19–21). Mackay et al. demonstrated that drugs with a positive charge of 10% show a significantly lower distribution than neutral drugs (20). Neutral or negatively charged drugs seem to optimally convect and distribute *via* CED. However, as negatively charged molecules have previously only been studied up to a charge of 10%, evidence of better convection and distribution of more negatively charged molecules is lacking. In addition, since CED circumvents the systemic circulation and thus the first pass effect, prodrugs are not suitable for local administration.

## Efficacy Simulation for Drug Delivery in DIPG

Table 1 shows the variables (i.e., the relevant physicochemical properties discussed above) included in the theoretical model and all drugs found in the literature search. The drugs were grouped based on their mechanisms of action (e.g., alkylating agents, topoisomerase inhibitors, signal transduction inhibitors, cytostatic antibiotics, antimitotic agents, antimetabolites, platinum-containing cytotoxics, antihormones, and others). An *“efficacy simulation*” was performed including the physicochemical properties of each chemotherapeutic agent and the environmental properties (i.e., pH 7.4). In this efficacy simulation every property was scored to be either optimal (1) or poor (0) based on the “Lipinski rule of five.” The sum of the scores for each variable was used to determine the likelihood of the drug to penetrate the BBB after systemic administration, or the likeliness of drug distribution over the tumor volume using CED. The colors in Table 1 indicate which chemotherapeutic agents are theoretically well suited for systemic and/or local administration *via* CED (score 3: marked green), or which chemotherapeutic agents are not (score 0/1: marked red). Chemotherapeutic agents with intermediate BBB penetration (score 2) have the potential to passively diffuse through the BBB, but likely in such low concentrations that they presumably do not reach therapeutic concentrations. These drugs are therefore marked yellow.

**Table 1:** Overview of the physicochemical properties of all chemotherapeutic drugs historically applied to DIPG patients.

Drug	Log *P*	Molecular weight (g/mol)	Charge (%)		Systemic delivery	Convection enhanced delivery
**Alkylating agents**						
Carmustine	1.53	214.10	0		+	+
Cyclophosphamide^a^	0.20	277.09	0		+/−	–
Dacarbazine^a^	−0.5	126.12	0		+/−	–
Ifosfamide^a^	0.20	277.09	0		+/−	–
Lomustine	2.83	233.70	0		+	–^b^
Melphalan	−0.5	305.20	99	(+/−)	–	–
Temozolomide	−1.1	194.15	0		+/−	–^b^
Topo-isomerase inhibitors						
Etoposide	0.60	588.56	1	(–)	+/−	+
Irinotecan^c^	3.50	586.70	100	(+)	+/−	–
Topotecan	−0.88	412.45	100	(+)	–	–
**Signal transduction inhibitors**						
Monoclonal antibodies						
Bevacizumab	Unknown	149,000.00	Unknown		–	–
Cetuximab	Unknown	145,781.60	Unknown		–	–
Nimotuzumab	Unknown	151,000.00	Unknown		–	–
Pembrolizumab	Unknown	146,286.29	Unknown		–	–
Tyrosine kinase inhibitors						
Afatinib	3.60	485.94	96	(+)	+/−	–
Cobimetinib	3.90	531.32	100	(+)	+/−	–
Crenolanib	3.70	443.54	100	(+)	+/−	–
Crizotinib	3.70	450.34	98	(+)	+/−	–
Dasatinib	3.60	488.01	40	(+)	+/−	–
Erlotinib	2.95	393.44	0		+	–^b^
Imatinib	3.25	493.60	88	(+)	+/−	–
Gefitinib	3.65	446.90	21	(+)	+/−	–^b^
Vandetanib	4.82	475.35	98	(+)	+/−	–
Proliferation signal inhibitors (mTOR)						
Everolimus	5.90	958.22	0		–	–^b^
Sirolimus	4.81	914.17	0		–	–^b^
Tacrolimus	3.30	804.02	0		+/−	+
Temsirolimus	4.25	1,030.29	0		–	+
Other signal transduction inhibitors						
Vismodegib	2.70	421.30	0		+	–^b^
**Cytotoxic antibiotics**						
Dactinomycin	3.21	1,255.42	99	(+)	–	–
Daunorubicin	1.83	527.52	98	(+)	–	–
Doxorubicin	1.28	543.51	98	(+)	–	–
Mitoxantrone	1.19	444.48	99	(+)	–	–
**Antimitotic agents**						
Cabazitaxel	2.70	835.93	0		+/−	+
Vincristine	2.82	824.96	98	(+)	–	–
Vinorelbine	4.84	778.93	100	(+)	–	–
**Antimetabolites**						
Capecitabine	0.56	359.35	12	(–)	+/−	–^b^
Cytarabine	−2.46	243.22	0		+/−	+
Gemcitabine	−2.01	263.19	0		+/−	+
Methotrexate	−1.85	454.44	100	(–)	–	+/−
**Platinum containing cytotoxics**						
Carboplatin	−0.19	371.25	0		+/−	+
Cisplatin	−2.19	300.05	0		+/−	+
**Antihormones**						
Tamoxifen	6.70	371.51	95	(+)	–	–
**Other chemotherapeutics**						
Abemaciclib	3.8	506.59	77	(+)	–	–
Cilengitide	−1	588.66	100	(+)	–	–
Imetelstat sodium	na	4,895.95	100	(+)	–	–
lenalidomide	−0.4	259.26	0		+	–^b^
Panobinostat	3.0	349.43	98	(+)	+/−	–
Ribociclib	2.2	434.54	96	(+)	+/−	–
Thalidomide	0.33	258.23	0		+	–^b^
Veliparib	0.5	244.29	99	(+)	+/−	–
Vorinostat	1.44	264.32	3	(–)	+	–^c^
**Other drugs**						
Mebendazole	2.83	295.29	8	(–)	+	–^b^
Valproic acid	2.75	144.21	99	(–)	+/−	+/−

*Green = drugs with good BBB penetration (systemic) or high distribution volume (CED); yellow = drugs with moderate BBB penetration (systemic) or moderate distribution volume (CED); red = drugs with limited BBB penetration (systemic) or limited distribution volume (CED)*.

*^a^Prodrug*.

*^b^Based on physicochemical properties, suitable for CED, however, only available for oral administration. Both drug and metabolite are active, drug does not penetrate the BBB and metabolite does*.

Drug affinity for efflux transporters (ATP-binding cassette (ABC) transporters) is an important factor in the prediction of ultimate brain uptake. These transporters include P-glycoprotein (P-gp/MDR1/ABCB1), breast cancer-resistant protein (BCRP/ABCG2), and multidrug resistance protein 1 (MRP1/ABCC1) (16, 17, 22). Drug affinity for these transporters has not been investigated for the majority of chemotherapeutics. Besides, as efflux transporter affinity strongly depends on the concentration of a drug, it is difficult to give uniform values on these transporters’ affinity. Therefore, the influence of ABC transporters could not be included in the model. For completeness, Table 2 was designed to summarize all *known* drug affinities for the efflux transporters located in the BBB.

**Table 2:** Overview of efflux transporter affinity of all chemotherapeutic drugs historically applied to DIPG patients.

	P-gp substrate	BCRP substrate	MRP1 substrate	References
**Alkylating agents**				
Carmustine	+	−	+	(6)
Cyclophosphamide	+	+	−	(23, 24)
Dacarbazine	+	na	na	(25)
Ifosfamide	+	na	+	(26, 27)
Lomustine	+	+	+	(28)
Melphalan	+	−	−	(6)
Temozolomide	+	+	−	(6, 29)
**Topo-isomerase inhibitors**				
Etoposide	+	+	+	(6)
Irinotecan	+	+	+	(30–32)
Topotecan	+	+	+	(33, 34)
**Signal transduction inhibitors**				
Monoclonal antibodies				
Bevacizumab	na	na	na	
Cetuximab	na	na	na	
Nimotuzumab	na	na	na	
Pembrolizumab	na	na	na	
Tyrosine kinase inhibitors				
Afatinib	+	+	na	(25)
Cobimetinib	+	−	na	(25)
Crenolanib	na	na	na	
Crizotinib	+	−	+	(35)
Dasatinib	+	+	+	(6, 36, 37)
Erlotinib	+	+	+	(6, 38, 39)
Imatinib	+	+	+	(37, 40, 41)
Gefitinib	+	+	+	(31, 39, 42, 43)
Vandetanib	+	+	−	(44–46)
Immosuppressives				
Everolimus	+	−	na	(47)
Sirolimus	+	+	na	(48, 49)
Tacrolimus	+	+	na	(49)
Temsirolimus	+	−	−	(6)
Other signal transduction inhibitors				
Vismodegib	+	na	na	(50, 51)
**Cytotoxic antibiotics**				
Dactinomycin	+	−	+	(52, 53)
Daunorubicin	+	+	+	(30)
Doxorubicin	+	+	+	(6)
Mitoxantrone	+	+	+	(6)
**Antimitotic agents**				
Cabazitaxel	+/−	−	na	(25)
Vincristine	+	−	+	(30–32)
Vinorelbine	+	na	−	(53–55)
**Antimetabolites**				
Capecitabine	na	na	na	
Cytarabine	+/−	−	na	(24, 53, 56)
Gemcitabine	+/−	na	+	(24, 57)
Methotrexate	+	+	+	(30, 31, 58)
**Platinum containing cytotoxics**				
Carboplatin	+	−	+	(6, 59)
Cisplatin	+/−	+	+	(23)
**Antihormones**				
Tamoxifen	+	+	−	(60–63)
**Other chemotherapeutics**				
Abemaciclib	+	+	na	
Cilengitide	+	na	na	(64)
Imetelstat sodium	na	na	na	
Lenalidomide	+	−	−	(25)
Panobinostat	−	na	−	(65)
Ribociclib	na	na	na	
Thalidomide	+/−	na	na	(66)
Veliparib	+	+	na	(67–69)
Vorinostat	na	na	na	(65)
**Other drugs**				
Mebendazole	−	na	na	(25)
Valproic acid	−	−	+	(70–72)

*na: not applicable*.

Based on our model, including 51 drugs, only 8 (15%)—carmustine, lomustine, erlotinib, vismodegib, lenalidomide, thalidomide, vorinostat, and mebendazole—appear to be of use for systemic administration (green cells in Table 1 column 5), presumably resulting in adequate brain uptake in case of an intact BBB. These drugs will very likely have good BBB passage due to their relatively small molecular weight, lipophilicity and limited molecular charge. Drugs expected to have limited BBB passage (marked in yellow) are either (partly) charged (e.g., dasatinib) or too hydrophilic (e.g., temozolomide), limiting their passive diffusion. Drugs that are marked in red are unlikely to penetrate an intact BBB, mostly due to their molecular charge in physiological environment (up to 100%).

Potential candidates for CED, listed green in Table 1 column 6, are carmustine, etoposide, tacrolimus, temsirolimus, cabazitaxel, cytarabine, gemcitabine, carboplatin, and cisplatin. These drugs have a neutral charge in physiological environment. Methotrexate and valproic acid (in yellow), being negatively charged, might also be suitable for CED, but this is speculative, as only molecules with a negative charge up to 10% have been investigated (20). The other drugs, marked in red, are theoretically not suitable for CED, mainly due to their positive charge (e.g., topotecan). Furthermore, prodrugs such as cyclophosphamide (indicated with superscript a) are not suitable for CED since these drugs require to be metabolized into their active (effective) metabolite. Additionally, drugs indicated with superscript b are currently only available for oral administration and not in liquid form. These drugs however are theoretically suitable for CED based on their favorable neutral charge. It might be worth to investigate reformulation of these drugs into liquid formula for local brain delivery applications.

## Discussion

In this study, we developed a theoretical model to review whether chemotherapeutics are suitable for passive diffusion through an intact BBB (after systemic administration) or whether local administration *via* convection-enhanced delivery (CED) may increase their therapeutic potential. We demonstrated that most systemically (intravenously or orally) administered chemotherapeutic drugs thus far investigated in clinical trials in DIPG are not likely to reach adequate intratumoral concentrations in case of intact BBB, rendering most of these therapies likely ineffective for use in DIPG.

To date, only few studies investigated the actual brain concentration of chemotherapeutic drugs after systemic administration. In 2007, Muldoon et al. reviewed these studies and showed that (from our list of chemotherapeutic drugs historically administered to DIPG patients) there was no brain uptake of doxorubicin, vincristine, and methotrexate after systemic administration in healthy and glioma-bearing dogs and rats (8). Cytarabine and etoposide showed low brain concentrations in these preclinical models. In adults, on-therapy biopsies showed low brain concentrations of cisplatin, imatinib gemcitabine, and methotrexate after systemic administration (8, 12–14). These results are in line with the results of our theoretical review.

Muldoon et al. also showed that the brain:plasma ratio found in preclinical and clinical pharmacology studies was variable, mainly due to the inconsistent interstitial fluid pressure and location of the samples taken, indicating heterogeneous drug distribution (8). Recently, we introduced PET imaging of zirconium-89 (^89^Zr)-labeled bevacizumab in children with DIPG and demonstrated considerable heterogeneity in drug delivery between patients and within DIPG tumors (73). PET technology enables imaging of radiolabeled drugs, especially monoclonal antibodies and tyrosine kinase inhibitors (74). By this non-invasive *in patient* quantification of tumor uptake and drug distribution not only therapeutic potential but also toxicity can be predicted. We advocate the development of molecular drug imaging studies additional or parallel to clinical trials because especially in children with cancer, drugs without therapeutic effect (based on a lack of drug-uptake in the tumor), may only cause (life-long) side effects.

In addition to the potential to reach therapeutic concentrations, a drug’s maximum tolerated dose and toxicity profile could be a limiting factor, even when a drug has proven to penetrate the BBB. Recent clinical studies in DIPG have, therefore, started focusing on alternative routes of drug delivery. Alternatives include CED, for which we here show that it theoretically increases the therapeutic potential of suited drugs previously administered systemically in DIPG patients without effect. It should be noted, however, that CED targets only the primary tumor site and will not target disseminated disease. As 13–17% of DIGP patients show distant parenchymal, subependymal, and leptomeningeal metastases in the brain and/or spine, CED should very likely be complemented with “whole-brain therapy” (75). Other alternative drug delivery techniques that are currently being investigated include (i) encapsulation of cationic substances into liposomes (micro- and nanoparticles) to decrease their tissue affinity and thus increase their volume of distribution and (ii) temporary BBB disruption techniques, such as focused or unfocused ultrasound-mediated drug delivery, to enhance uptake of systemically delivered drugs (19–21, 76). Since the variables that determine local drug concentrations in these techniques are heterogeneous and not widely investigated yet, these alternative techniques could not be taken into account in our model design and review. The exact extent and timing of radiotherapy-induced BBB disruption in DIPG is unknown and warrants further investigation.

In conclusion, this review raises awareness for the impact of physicochemical properties of anticancer drugs that influence their passive diffusion through an intact BBB after systemic administration. In diffuse gliomas such as DIPG, in which the BBB is largely intact in large parts of the tumor, during most time of the disease course, one must critically weigh drug candidates that *a priori* are unlikely to pass the BBB and thus are unlikely to have therapeutic effect in patients suffering from these tumors. This might also be valid for other diffuse growing brain tumors in child- and adulthood that show areas of tumor infiltration behind an intact BBB. We furthermore postulate that a novel drug delivery technique, such as CED theoretically increases the therapeutic potential of some of the drugs previously administered systemically. This might require repositioning of these drugs and reformulation to render them suitable for local delivery strategies.

## Future Prospects

Our review calls for further preclinical BBB drug delivery models, as well as clinical research on actual intratumoral drug uptake and local drug delivery techniques such as CED. The model itself may be helpful in the design of future treatment regimens in which the combination of systemic administration and local or alternative delivery of different chemotherapeutics is explored. It aims to provide a first selection of drugs that have the highest potential to penetrate the BBB. Ultimately, best (combinations) of potentially effective drugs against DIPG can be sought combining these data with IC50 data from preclinical studies (77), and information on drug efflux mechanisms. Ideally, this theoretical BBB-passage model needs (pre)clinical validation. Although research into intratumoral drug concentrations remains challenging, especially in DIPG, a preclinical validation study of our model is currently being developed. Clinical validation using on-therapy tumor biopsy studies (i.e., to directly measure intratumoral drug uptake after systemic administration of chemotherapeutic agents), or less invasively with PET imaging (73) are currently emerging and will provide further information on drug uptake in these detrimental diseases.

## Author Contributions

SVZ and FE contributed to the design of the work, data collection and interpretation, and wrote the main body of the manuscript. DV contributed to the interpretation of the data and critically revised the manuscript. TS contributed to the interpretation of the data. EH assisted in data collection. NH contributed to the interpretation of the data and revised the manuscript. GK critically revised the manuscript and assisted in writing the manuscript.

## Conflict of Interest Statement

The authors declare that the research was conducted in the absence of any commercial or financial relationships that could be construed as a potential conflict of interest.
