# Supplementary material for: Effective Drug Delivery in Diffuse Intrinsic Pontine Glioma: A Theoretical Model to Identify Potential Candidates
Source: Front Oncol. 2017 Oct 30;7:254. doi: 10.3389/fonc.2017.00254 (PMC5670105; doi:10.3389/fonc.2017.00254)
Supplement: Supplementary file 1 [file data_sheet_1.docx]

**Supplementary search strategy**

PubMed

**#1 Pontine gliomas**

"Diffuse Intrinsic Pontine Gliomas"[tiab] OR "Diffuse Intrinsic Pontine Glioma"[tiab] OR DIPG[tiab] OR "Brain Stem Neoplasms"[Mesh] OR (("Pons"[Mesh] OR pons[tiab] OR pontine[tiab]) AND ("Neoplasms"[Mesh] OR Neoplasm[tiab] OR neoplasms[tiab] OR neoplasia[tiab] OR neoplasma[tiab] OR neoplasmatic[tiab] OR neoplastic[tiab] OR cancer[tiab] OR cancers[tiab] OR cancerous[tiab] OR cancereux[tiab] OR canceration[tiab] OR cancerisation[tiab] OR cancerization[tiab] OR cancerogen*[tiab] OR cancerology[tiab] OR cancerologie[tiab] OR precancerous[tiab] OR tumour[tiab] OR tumor[tiab] OR tumours[tiab] OR tumors[tiab] OR tumourous[tiab] OR tumorous[tiab] OR carcinom*[tiab] OR glioma*[tiab]))

NOT ("addresses"[Publication Type] OR "biography"[Publication Type] OR  "comment"[Publication Type] OR "directory"[Publication Type] OR "editorial"[Publication Type] OR "festschrift"[Publication Type] OR "interview"[Publication Type] OR "lectures"[Publication Type] OR "legal cases"[Publication Type] OR "legislation"[Publication Type] OR "letter"[Publication Type] OR "news"[Publication Type] OR "newspaper article"[Publication Type] OR "patient education handout"[Publication Type] OR "popular works"[Publication Type] OR "congresses"[Publication Type] OR "consensus development conference"[Publication Type] OR "consensus development conference, nih"[Publication Type] OR "practice guideline"[Publication Type])

| **Search** | **PubMed Query** | **Items found** |
| --- | --- | --- |
| [**#2**](http://www.ncbi.nlm.nih.gov/pubmed) | #1 NOT ("addresses"[Publication Type] OR "biography"[Publication Type] OR "comment"[Publication Type] OR "directory"[Publication Type] OR "editorial"[Publication Type] OR "festschrift"[Publication Type] OR "interview"[Publication Type] OR "lectures"[Publication Type] OR "legal cases"[Publication Type] OR "legislation"[Publication Type] OR "letter"[Publication Type] OR "news"[Publication Type] OR "newspaper article"[Publication Type] OR "patient education handout"[Publication Type] OR "popular works"[Publication Type] OR "congresses"[Publication Type] OR "consensus development conference"[Publication Type] OR "consensus development conference, nih"[Publication Type] OR "practice guideline"[Publication Type]) | [**3039**](http://www.ncbi.nlm.nih.gov/pubmed/?cmd=HistorySearch&querykey=25) |
| **#1** | "Diffuse Intrinsic Pontine Gliomas"[tiab] OR "Diffuse Intrinsic Pontine Glioma"[tiab] OR DIPG[tiab] OR "Brain Stem Neoplasms"[Mesh] OR (("Pons"[Mesh] OR pons[tiab] OR pontine[tiab]) AND ("Neoplasms"[Mesh] OR Neoplasm[tiab] OR neoplasms[tiab] OR neoplasia[tiab] OR neoplasma[tiab] OR neoplasmatic[tiab] OR neoplastic[tiab] OR cancer[tiab] OR cancers[tiab] OR cancerous[tiab] OR cancereux[tiab] OR canceration[tiab] OR cancerisation[tiab] OR cancerization[tiab] OR cancerogen*[tiab] OR cancerology[tiab] OR cancerologie[tiab] OR precancerous[tiab] OR tumour[tiab] OR tumor[tiab] OR tumours[tiab] OR tumors[tiab] OR tumourous[tiab] OR tumorous[tiab] OR carcinom*[tiab] OR glioma*[tiab])) | [**3160**](http://www.ncbi.nlm.nih.gov/pubmed/?cmd=HistorySearch&querykey=14) |

**#2 Pediatric High Grade Glioma**

("Glioma"[Mesh] OR glioma*[tiab]) AND ("Infant"[Mesh] OR "Child"[Mesh] OR child[tiab] OR children[tiab] OR children’s[tiab] childrens[tiab] OR childhood[tiab] OR schoolchild*[tiab] OR infan*[tiab] OR adolescen*[tiab] OR pediatri*[tiab] OR paediatr*[tiab] OR neonat*[tiab] OR boy[tiab] OR boys[tiab] OR boyhood[tiab] OR girl[tiab] OR girls[tiab] OR girlhood[tiab] OR youth[tiab] OR youths[tiab] OR baby[tiab] OR babies[tiab] OR toddler*[tiab] OR teen[tiab] OR teens[tiab] OR teenager*[tiab] OR newborn*[tiab] OR postneonat*[tiab] OR postnat*[tiab] OR puberty[tiab] OR preschool*[tiab] OR suckling*[tiab] OR picu[tiab] OR nicu[tiab] OR offspring[tiab] OR minor[tiab] OR minors[tiab])

NOT ("addresses"[Publication Type] OR "biography"[Publication Type] OR  "comment"[Publication Type] OR "directory"[Publication Type] OR "editorial"[Publication Type] OR "festschrift"[Publication Type] OR "interview"[Publication Type] OR "lectures"[Publication Type] OR "legal cases"[Publication Type] OR "legislation"[Publication Type] OR "letter"[Publication Type] OR "news"[Publication Type] OR "newspaper article"[Publication Type] OR "patient education handout"[Publication Type] OR "popular works"[Publication Type] OR "congresses"[Publication Type] OR "consensus development conference"[Publication Type] OR "consensus development conference, nih"[Publication Type] OR "practice guideline"[Publication Type])

| **Search** | **PubMed Query** | **Items found** |
| --- | --- | --- |
| #2 | #2 NOT ("addresses"[Publication Type] OR "biography"[Publication Type] OR "comment"[Publication Type] OR "directory"[Publication Type] OR "editorial"[Publication Type] OR "festschrift"[Publication Type] OR "interview"[Publication Type] OR "lectures"[Publication Type] OR "legal cases"[Publication Type] OR "legislation"[Publication Type] OR "letter"[Publication Type] OR "news"[Publication Type] OR "newspaper article"[Publication Type] OR "patient education handout"[Publication Type] OR "popular works"[Publication Type] OR "congresses"[Publication Type] OR "consensus development conference"[Publication Type] OR "consensus development conference, nih"[Publication Type] OR "practice guideline"[Publication Type]) | 6681 |
| #1 | ("Glioma"[Mesh] OR glioma*[tiab]) AND ("Infant"[Mesh] OR "Child"[Mesh] OR child[tiab] OR children[tiab] OR children’s[tiab] childrens[tiab] OR childhood[tiab] OR schoolchild*[tiab] OR infan*[tiab] OR adolescen*[tiab] OR pediatri*[tiab] OR paediatr*[tiab] OR neonat*[tiab] OR boy[tiab] OR boys[tiab] OR boyhood[tiab] OR girl[tiab] OR girls[tiab] OR girlhood[tiab] OR youth[tiab] OR youths[tiab] OR baby[tiab] OR babies[tiab] OR toddler*[tiab] OR teen[tiab] OR teens[tiab] OR teenager*[tiab] OR newborn*[tiab] OR postneonat*[tiab] OR postnat*[tiab] OR puberty[tiab] OR preschool*[tiab] OR suckling*[tiab] OR picu[tiab] OR nicu[tiab] OR offspring[tiab] OR minor[tiab] OR minors[tiab]) | 6791 |

EMBASE

**#1 Pontine gliomas**

'Diffuse Intrinsic Pontine Gliomas':ti,ab OR 'Diffuse Intrinsic Pontine Glioma':ti,ab OR DIPG:ti,ab OR 'brain stem tumor'/exp OR ('pons'/exp OR pons:ti,ab OR pontine:ti,ab) AND ('neoplasm'/exp OR Neoplasm:ti,ab OR neoplasms:ti,ab OR neoplasia:ti,ab OR neoplasma:ti,ab OR neoplasmatic:ti,ab OR neoplastic:ti,ab OR cancer:ti,ab OR cancers:ti,ab OR cancerous:ti,ab OR cancereux:ti,ab OR canceration:ti,ab OR cancerisation:ti,ab OR cancerization:ti,ab OR cancerogen*:ti,ab OR cancerology:ti,ab OR cancerologie:ti,ab OR precancerous:ti,ab OR tumour:ti,ab OR tumor:ti,ab OR tumours:ti,ab OR tumors:ti,ab OR tumourous:ti,ab OR tumorous:ti,ab OR carcinom*:ti,ab OR glioma*:ti,ab)

AND ('Article'/it OR 'Article in Press'/it OR 'Review'/it)

## Embase Session Results

| No. | Query | Results |
| --- | --- | --- |
| #2 | **#1** AND (**'article'**/it OR **'article in press'**/it OR **'review'**/it) | **3224** |
| #1 | **'diffuse intrinsic pontine gliomas'**:ab,ti OR **'diffuse intrinsic pontine glioma'**:ab,ti OR **dipg**:ab,ti OR **'brain stem tumor'**/exp OR **'pons'**/exp OR **pons**:ab,ti OR **pontine**:ab,ti AND (**'neoplasm'**/exp OR **neoplasm**:ab,ti OR **neoplasms**:ab,ti OR **neoplasia**:ab,ti OR **neoplasma**:ab,ti OR **neoplasmatic**:ab,ti OR **neoplastic**:ab,ti OR **cancer**:ab,ti OR **cancers**:ab,ti OR **cancerous**:ab,ti OR **cancereux**:ab,ti OR **canceration**:ab,ti OR **cancerisation**:ab,ti OR **cancerization**:ab,ti OR **cancerogen***:ab,ti OR **cancerology**:ab,ti OR **cancerologie**:ab,ti OR **precancerous**:ab,ti OR **tumour**:ab,ti OR **tumor**:ab,ti OR **tumours**:ab,ti OR **tumors**:ab,ti OR **tumourous**:ab,ti OR **tumorous**:ab,ti OR **carcinom***:ab,ti OR **glioma***:ab,ti) | **4327** |

**#2 Pediatric High Grade Glioma**

('glioma'/exp OR glioma*:ti,ab) AND ('child'/exp OR 'newborn'/exp OR child:ti,ab OR children*:ti,ab OR childhood:ti,ab OR schoolchild*:ti,ab OR infan*:ti,ab OR adolescen*:ti,ab OR pediatri*:ti,ab OR paediatr*:ti,ab OR neonat*:ti,ab OR boy:ti,ab OR boys:ti,ab OR boyhood:ti,ab OR girl:ti,ab OR girls:ti,ab OR girlhood:ti,ab OR youth:ti,ab OR youths:ti,ab OR baby:ti,ab OR babies:ti,ab OR toddler*:ti,ab OR teen:ti,ab OR teens:ti,ab OR teenager*:ti,ab OR newborn*:ti,ab OR postneonat*:ti,ab OR postnat*:ti,ab OR puberty:ti,ab OR preschool*:ti,ab OR suckling*:ti,ab OR picu:ti,ab OR nicu:ti,ab OR offspring:ti,ab OR minor:ti,ab OR minors:ti,ab)

AND ('Article'/it OR 'Article in Press'/it OR 'Review'/it)



## Embase Session Results

| No. | Query | Results |
| --- | --- | --- |
| #9 | **#8** AND (**'article'**/it OR **'article in press'**/it OR **'review'**/it) | **13531** |
| #8 | **'glioma'**/exp OR **glioma***:ab,ti AND (**'child'**/exp OR **'newborn'**/exp OR **child**:ab,ti OR **children***:ab,ti OR **childhood**:ab,ti OR **schoolchild***:ab,ti OR **infan***:ab,ti OR **adolescen***:ab,ti OR **pediatri***:ab,ti OR **paediatr***:ab,ti OR **neonat***:ab,ti OR **boy**:ab,ti OR **boys**:ab,ti OR **boyhood**:ab,ti OR **girl**:ab,ti OR **girls**:ab,ti OR **girlhood**:ab,ti OR **youth**:ab,ti OR **youths**:ab,ti OR **baby**:ab,ti OR **babies**:ab,ti OR **toddler***:ab,ti OR **teen**:ab,ti OR **teens**:ab,ti OR **teenager***:ab,ti OR **newborn***:ab,ti OR **postneonat***:ab,ti OR **postnat***:ab,ti OR **puberty**:ab,ti OR **preschool***:ab,ti OR **suckling***:ab,ti OR **picu**:ab,ti OR **nicu**:ab,ti OR **offspring**:ab,ti OR **minor**:ab,ti OR **minors**:ab,ti) | **17655** |

Cochrane Library

**#1 Pontine gliomas**

"Diffuse Intrinsic Pontine Gliomas" OR "Diffuse Intrinsic Pontine Glioma" OR DIPG OR "Brain Stem Neoplasms" OR ((pons OR pontine) AND (Neoplasm OR neoplasms OR neoplasia OR neoplasma OR neoplasmatic OR neoplastic OR cancer OR cancers OR cancerous OR cancereux OR canceration OR cancerisation OR cancerization OR cancerogen* OR cancerology OR cancerologie OR precancerous OR tumour OR tumor OR tumours OR tumors OR tumourous OR tumorous OR carcinom* OR glioma*))

| **No.** | **Cochrane query** | Results |
| --- | --- | --- |
| #1 | "Diffuse Intrinsic Pontine Gliomas" or "Diffuse Intrinsic Pontine Glioma" or DIPG or "Brain Stem Neoplasms" or ((pons or pontine) and (Neoplasm or neoplasms or neoplasia or neoplasma or neoplasmatic or neoplastic or cancer or cancers or cancerous or cancereux or canceration or cancerisation or cancerization or cancerogen* or cancerology or cancerologie or precancerous or tumour or tumor or tumours or tumors or tumourous or tumorous or carcinom* or glioma*)):ti,ab,kw | 15 |

**#2 Pediatric High Grade Glioma**

glioma* AND (child OR children OR children’s childrens OR childhood OR schoolchild* OR infan* OR adolescen* OR pediatri* OR paediatr* OR neonat* OR boy OR boys OR boyhood OR girl OR girls OR girlhood OR youth OR youths OR baby OR babies OR toddler* OR teen OR teens OR teenager* OR newborn* OR postneonat* OR postnat* OR puberty OR preschool* OR suckling* OR picu OR nicu OR offspring OR minor OR minors)

| **No.** | **Cochrane query** | Results |
| --- | --- | --- |
| #2 | glioma* and (child or children or children’s childrens or childhood or schoolchild* or infan* or adolescen* or pediatri* or paediatr* or neonat* or boy or boys or boyhood or girl or girls or girlhood or youth or youths or baby or babies or toddler* or teen or teens or teenager* or newborn* or postneonat* or postnat* or puberty or preschool* or suckling* or picu or nicu or offspring or minor or minors):ti,ab,kw | 134 |
